# Strigolactones and Shoot Branching: What Is the Real Hormone and How Does It Work?

**DOI:** 10.1093/pcp/pcad088

**Published:** 2023-08-01

**Authors:** Elizabeth A Dun, Philip B Brewer, Elizabeth M J Gillam, Christine A Beveridge

**Affiliations:** ARC Centre of Excellence for Plant Success in Nature and Agriculture, The University of Queensland, St Lucia, QLD 4072, Australia; School of Agriculture and Food Sustainability, The University of Queensland, St Lucia, QLD 4072, Australia; ARC Centre of Excellence for Plant Success in Nature and Agriculture, The University of Queensland, St Lucia, QLD 4072, Australia; Waite Research Institute, School of Agriculture Food & Wine, The University of Adelaide, Adelaide, SA 5064, Australia; School of Chemistry and Molecular Biosciences, The University of Queensland, St Lucia, QLD 4072, Australia; ARC Centre of Excellence for Plant Success in Nature and Agriculture, The University of Queensland, St Lucia, QLD 4072, Australia; School of Agriculture and Food Sustainability, The University of Queensland, St Lucia, QLD 4072, Australia

**Keywords:** Bud outgrowth, Shoot branching, Strigolactone biosynthesis, Strigolactone signaling, Strigolactone transport, Tillering

## Abstract

There have been substantial advances in our understanding of many aspects of strigolactone regulation of branching since the discovery of strigolactones as phytohormones. These include further insights into the network of phytohormones and other signals that regulate branching, as well as deep insights into strigolactone biosynthesis, metabolism, transport, perception and downstream signaling. In this review, we provide an update on recent advances in our understanding of how the strigolactone pathway co-ordinately and dynamically regulates bud outgrowth and pose some important outstanding questions that are yet to be resolved.

## Introduction

Axillary buds are located in the axils of leaves and consist of an axillary meristem protected by surrounding leaf primordia and young leaves. Throughout the life of a plant, the fate of each axillary bud is highly regulated. Whether a bud remains inhibited or develops into a branch/tiller depends on intricate signaling networks that integrate environmental and genetic factors. The differential regulation of the fate of these axillary buds contributes to the amazing plasticity observed in plant shoot architectures, even in genetically identical plants. Understanding this regulation and the genetic control of shoot branching is crucial as bud outgrowth is an important agronomic trait that contributes to the overall shoot architecture of a plant and is a potential target for yield optimization in diverse food, ornamental and forestry crops (Chesterfield et al. 2020, Kelly et al. 2023).

**Fig. 1:**
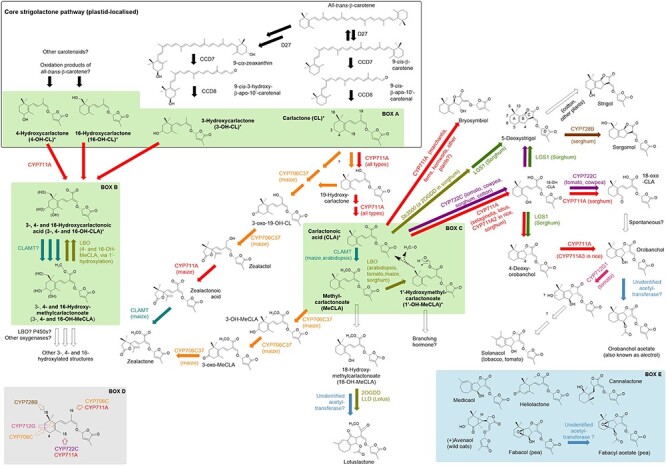
The SL biosynthesis pathway highlighting the involvement of known enzymes and intermediates. The plastid-localized core SL pathway commences with all-*trans*-β-carotene. Initial reactions to produce CL and hydroxylated CLs (OH-CLs) are relatively conserved among plants. Subsequent reactions lead to a huge diversity of SL structures both within and among species. Canonical SLs have an ABCD-ring structure (as indicated on 5-deoxystrigol), while non-canonical SLs lack either the B- or C-ring. Much of the diversity in structure is due to rearrangement and decoration of the CL scaffold. Boxes A, B and C highlight SLs and precursors (in bold text) that have been detected in shoot tissue and are therefore likely important for the production of the SL shoot branching hormone. While 5-deoxystrigol has been detected in shoot tissue, it is at the limit of detection. *Denotes SLs and precursors demonstrated by grafting to likely move long distance throughout the plant in the direction of rootstock to shoot, presumably in the xylem (CL, 3-, 4-, 16-OH-CL, CLA, 3-, 4-, 16-OH-CLA, 1ʹ-OH-MeCLA). CLA (Box C) is unlikely to traverse membranes while in the shoot due to the presence of a charged carboxylate group. MeCLA is neutral and so likely more membrane permeable than CLA. Box D highlights the regioselectivity of the P450 enzyme families. Box E shows SLs with enzymes unknown. Production of orobanchyl acetate and fabacyl acetate has not yet been determined but is likely to involve a currently unidentified acetyl transferase enzyme. Arrows are color-coded to represent different enzyme families, including 2OGDDs, and are labeled with enzyme names and species where relevant. Solid arrows represent reactions that have been demonstrated, white (empty) arrows represent reactions with as yet unidentified catalysts and dotted arrows indicate spontaneous conversions. Ring numbering is indicated on the CL and 5-deoxystrigol structures.

Strigolactones (SLs) are the most recently identified of a number of phytohormones that affect shoot branching (Beveridge et al. 2023). SLs are a group of carotenoid-derived molecules of related but diverse structure that contain a methylbutenolide ring that is critical for bioactivity in bud outgrowth inhibition (Umehara et al. 2015, Waters et al. 2017) (**Fig. 1**). They were originally identified as important plant-derived rhizosphere signaling molecules that function as parasitic plant seed germination stimulants (Cook et al. 1972) and were later demonstrated to promote beneficial arbuscular mycorrhizal symbioses (Akiyama et al. 2005). Broadly speaking, SLs are now considered as plant hormones that regulate various aspects of plant growth and development *in planta*, including bud outgrowth, plant height, senescence, adventitious and lateral root growth and root hair development (Rehman et al. 2021). It is likely that the ancestral role for SLs was as rhizosphere signaling molecules and that they were later recruited as plant hormones. This is because the bryophyte *Marchantia paleacea* lacks the ability to respond to SLs, but secretes bryosymbiol, an ancestral SL that is also present in vascular plants (Kodama et al. 2022) (**Fig. 1**).

The functional significance of the diversity of SLs found within and among plant species is only starting to be elucidated (e.g. Koichi Yoneyama et al. 2018, Yoneyama and Brewer 2021, Ito et al. 2022, Chen et al. 2023). SLs can be classified into two categories based on their chemical structure: canonical SLs and non-canonical SLs. While canonical SLs possess a tricyclic lactone (ABC-ring) connected via an enol–ether bond to the methylbutenolide D-ring, non-canonical SLs lack either the B or C ring (reviewed in Yoneyama and Brewer 2021, Kelly et al. 2023) (**Fig. 1**). Over 35 SLs have been so far identified (Bouwmeester et al. 2003, Chesterfield et al. 2020, Mashiguchi et al. 2021, Koichi Yoneyama et al. 2018) and show diversity in their distribution across plant species.

## SL Biosynthesis—Core and Canonical Pathways

The SL biosynthesis pathway has been deduced by a combination of physiological, biochemical and genetic studies using mutants with increased branching and reverse genetics in various species including *Petunia hybrida* (petunia), *Pisum sativum* (pea), *Arabidopsis thaliana* (arabidopsis) and *Oryza sativa* (rice) (reviewed in Mashiguchi et al. 2021, Beveridge et al. 2023). As it is currently understood, the SL biosynthesis pathway is conserved for initial steps to carlactone (CL), named the core pathway, before diverging to produce distinctive SLs (**Fig. 1**). As part of this core pathway, sequential reactions in the plastid result in the production of CL from all-*trans*-β-carotene (Seto et al. 2014, Jia et al. 2018, Mashiguchi et al. 2021) (**Fig. 1**). Plastid-localized β-carotene isomerase/DWARF27 (D27) catalyzes the isomerization of all-*trans*-β-carotene into 9-*cis*-β-carotene (Lin et al. 2009), which then undergoes sequential cleavage and rearrangement by CAROTENOID CLEAVAGE DIOXYGENASE 7 (CCD7) and CCD8 to produce CL (Alder et al. 2012). Interestingly, the core SL biosynthesis pathway, consisting of D27, CCD7 and CCD8, can produce other novel CL-like products such as 3-hydroxy-CL (3-OH-CL) *in planta* (Baz et al. 2018) (**Fig. 1**, Box A), and these hydroxylated CLs might be further converted to hydroxylated carlactonoic acids (CLAs) and methyl carlactonoates (MeCLAs)/novel SLs (Yoneyama et al. 2020a) (**Fig. 1**, Box B). The role of these SLs is yet to be established and, as discussed later, may be important for SL regulation of shoot branching.

CL and hydroxylated CLs are the universal precursors to all known SLs and are the central point from which the biosynthetic pathway diverges to produce the variety of canonical and non-canonical SLs found across the plant kingdom. Mutation of any of the known SL biosynthetic enzymes required to produce CL (and hydroxylated CLs) results in plants with an increased branching or tillering phenotype (Brewer et al. 2013). As discussed later, the diversity in endogenous SL structures compared with branching phenotypes has not yet led to clarity around the bioactive SL in shoot branching.

Much of the structural diversity in SLs is due to rearrangement and decoration of the CL scaffold by various combinations of cytochrome P450 enzymes (P450s or CYPs; **Fig. 1**), a large and almost ubiquitously distributed family of hemoprotein monooxygenases that introduce structural diversity into organic molecules in many biological contexts. P450s use molecular oxygen and a reducing cofactor, typically NADPH, to insert one oxygen atom into a substrate and reduce the other to water. In so doing, they can accomplish a diverse range of biotransformation reactions including hydroxylations, dealkylations, ring closures and rearrangements (Guengerich 2001). P450s require the help of one or more shared redox partners to transfer the electrons from NADPH to the heme prosthetic group in the P450 active site. In the case of most plant P450s, this redox partner is a diflavin oxidoreductase enzyme, NADPH-cytochrome P450 reductase. Since the catalytic versatility of P450s stems from the heme prosthetic group, a given P450 can catalyze many different types of chemistry and often on many different substrates. Therefore, they cannot usefully be classified based on function alone (Nebert et al. 1987). Rather, P450s are classified based on amino acid sequence identity into families (>40% sequence identity; indicated by a number after the ‘CYP’ prefix, e.g. CYP711) and subfamilies (>55% amino acid identity; indicated by a subsequent letter, e.g. CYP711A). Individual forms within a given subfamily are indicated by a final number (e.g. CYP711A1) (Nelson et al. 1993).

CL is oxidized to produce CLA by CYP711A1 in arabidopsis [also known as MORE AXILLARY GROWTH1 (MAX1)] and CYP711A subfamily homologs in many other species (Abe et al. 2014, Kaori Yoneyama et al. 2018, Mori et al. 2020b) (**Fig. 1**). For example, four of the five identified rice cytochrome P450 CYP711A subfamily members can catalyze conversion of CL to CLA; the fifth sequence has a premature stop codon and encodes an incomplete, non-functional protein (Challis et al. 2013, Zhang et al. 2014, Kaori Yoneyama et al. 2018, Marzec et al. 2020).

Functional diversity of CYP711A subfamily members and P450 enzymes from at least four other subfamilies, CYP706C, CYP712G, CYP722C and CYP728B, contribute to differential SL production during the subsequent steps of the canonical SL biosynthesis pathway downstream of CL, whereby distinct reactions to produce different SLs are catalyzed by different but related enzymes (Wu et al., 2023, Koichi Yoneyama et al. 2018, Zhang et al. 2018, Wakabayashi et al. 2019, 2020, 2021, Marzec et al. 2020, Mori et al. 2020b, 2020a, Y. Wang et al. 2022, Sigalas et al. 2023) (**Fig. 1**). For example, in *Lotus japonicus* CL is converted to 18-hydroxy CLA (18-OH-CLA) via CLA by CYP711A9 and 18-OH-CLA is converted to 5-deoxystrigol by CYP722C, encoded by the gene *5-DEOXYSTRIGOL DEFECTIVE* (*DSD*) (Mori et al. 2020a, 2020b). However, in *Solanum lycopersicum* (tomato) and *Vigna unguiculata* (cowpea), CLA is converted to orobanchol (via intermediates) by CYP722C (Wakabayashi et al. 2019). In contrast, in *Gossypium arboreum* (cotton), CYP722C converts CLA to 5-deoxystrigol via 18-OH-CLA (Wakabayashi et al. 2020).

Other enzymes are involved in the canonical SL biosynthesis pathway, including a sulfotransferase that, as discussed later, determines the stereochemistry of SLs produced (Gobena et al. 2017, Wu and Li 2021, Yoda et al. 2021) (**Fig. 1**). Based on identified SL structures, other enzymes and pathways remain to be characterized. For example, an as-yet unidentified acetyl transferase is likely to be involved in the biosynthetic pathways of two major SLs in the root exudate of pea, fabacyl acetate and orobanchyl acetate (Yoneyama et al. 2008, Xie et al. 2009) (**Fig. 1**). In contrast with the vast bulk of enzymes identified to date, the discovery of additional enzymes is unlikely to come from a forward genetics approach (Beveridge et al. 2023) because the branching phenotype screens of mutagenized populations appear to be at saturation (Johnson et al. 2006). Hence, approaches that do not primarily rely on phenotypic screens, such as reverse genetics based on enzymatic function or co-expression analysis, may be increasingly required, as used for the discovery of LATERAL BRANCHING OXIDOREDUCTASE (LBO) and CLA-methyltransferase (CLAMT) discussed later (Brewer et al. 2016, Mashiguchi et al. 2022).

## Canonical SLs—Unlikely the Shoot Branching Hormone *in planta*?

New findings question the importance of canonical SLs for the regulation of bud outgrowth *in planta*. Although exogenous treatment of canonical SLs 5-deoxystrigol, 4-deoxyorobanchol, orobanchol or solanacol (and others) can inhibit bud outgrowth (e.g. Umehara et al. 2008, Boyer et al. 2012, Scaffidi et al. 2014), this does not mean that these SLs control shoot branching *in planta*. The typical increased branching phenotype expected of SL biosynthesis mutant plants is not observed in the tomato *slcyp722c*-knockout mutant (Wakabayashi et al. 2019), *L. japonicus cyp711a9* mutant (Mori et al. 2020b), rice *oscyp711a2*-knockout mutant (Ito et al. 2022) or rice *oscyp711a2 oscyp711a3* double mutants (Chen et al. 2023) despite particular canonical SLs being undetectable in the root and/or exudate (Wakabayashi et al. 2019, Mori et al. 2020b, Ito et al. 2022, Chen et al. 2023). These canonical SLs that were deficient in these non-branching plants were orobanchol and solanacol (tomato), 5-deoxystrigol (and the non-canonical SL, lotuslactone; lotus), 4-deoxyorobanchol and orobanchol (rice). Combined, these results suggest that canonical SLs are not the shoot branching hormone, nor are they required for the production of the shoot branching hormone. Indeed, as discussed later, canonical SLs are not detectable in shoot tissue (Yoneyama et al. 2007b, Umehara et al. 2010; Xie et al. 2015b, Ito et al. 2022) or are at the limit of detection (Yoneyama et al. 2007b, Umehara et al. 2010).

There is precedence for specific SL molecular structures having differing functions, as different canonical SLs in the root exudate have different activity toward parasitic weed seed germination, and the composition of SL molecular structures in the root exudate is under genetic control. For example, in sorghum (*Sorghum bicolor*), a sulfotransferase LOW GERMINATION STIMULANT1 (LGS1) functions together with a 2-oxoglutarate-dependent dioxygenase (2OGDD) to determine the dominant SL molecular structure in the root exudate (Gobena et al. 2017, Yoda et al. 2023). Orobanchol is the dominant SL in sorghum *lgs1* mutant plant root exudates, instead of 5-deoxystrigol, and this corresponds to low parasitic weed seed germination stimulant activity of mutant exudate but no observed increase in tillering (Gobena et al. 2017) (**Fig. 1**). Additionally, *in silico* analyses revealed that the five CYP711A genes in rice vary widely in the regulation of their expression, suggesting that there may be differences in function for different rice SLs produced by those enzymes and that the regulation of the production of specific SLs is possible (Marzec et al. 2020). It is therefore plausible that spatial localization of specific SL molecular structures *in planta* is a key regulated process and that SL production in the root and root exudate is specific to the roles of SLs in the rhizosphere and independent of *in planta* hormonal functions of SLs. Another possibility, similar to animal systems, is that different SL-regulated processes in the shoot are regulated by different SLs that induce different signaling outcomes despite the same receptor (Hall et al. 2001, Smith et al. 2018).

## Non-canonical SL Biosynthesis—Are We Close to Identifying the Shoot Branching Hormone?

One pathway to the production of non-canonical SLs starts with the conversion of CLA to MeCLA by CLAMT (Mashiguchi et al. 2022, Li et al. 2023) (**Fig. 1**). Unlike mutants in the canonical SL pathway downstream of CLA, *clamt* loss-of-function mutants have moderately increased branching along with an accumulation of CLA and reduced MeCLA (Mashiguchi et al. 2022). This supports the role of non-canonical SLs produced downstream of CLA in the inhibition of bud outgrowth. It is likely that the intermediate increased branching phenotype of *clamt* is due to the low but detectable levels of MeCLA, indicating possible enzymatic redundancy (Mashiguchi et al. 2022).

MeCLA is metabolized by LBO, a 2OGDD, into hydroxymethyl carlactonoate (1ʹ-OH-MeCLA; **Fig. 1**) (Brewer et al. 2016, Yoneyama et al. 2020a). However, 1ʹ-OH-MeCLA is chemically unstable and is likely converted rapidly back to CLA with the elimination of formaldehyde (**Fig. 1**). This activity has been attributed to LBO but is probably simply a consequence of the production of 1ʹ-OH-MeCLA. Similarly, 4-OH-MeCLA and 16-OH-MeCLA are converted back to their corresponding hydroxylated CLA and this has been attributed to LBO (**Fig. 1**, Box B) (Yoneyama et al. 2020a). Further research is required to determine if hydroxylated MeCLAs can be metabolized by LBO into other hydroxylated structures (**Fig. 1**, Box B). LBO has been demonstrated as functionally relevant in arabidopsis with increased branching observed in *lbo* mutants (Brewer et al. 2016). The identification of tomato, maize (*Zea mays*) and sorghum LBO homologs that can perform the same reaction in protein assays opens the pathway to reverse genetics approaches (Yoneyama et al. 2020a). 1ʹ-OH-MeCLA (and other hydroxylated structures downstream of hydroxylated MeCLAs) is a candidate for the endogenous SL branching hormone (**Fig. 1**, Boxes B and C). The increased lability of this and other non-canonical SLs relative to canonical SLs makes isolation difficult (Koichi Yoneyama et al. 2018), so it is not yet known if 1ʹ-OH-MeCLA is further converted to a downstream product that functions as the endogenous SL branching hormone (Yoneyama et al. 2020a) (**Fig. 1**). While *in vitro* assays demonstrate that MeCLA is a substrate for LBO and 1ʹ-OH-MeCLA is a reaction product, CLA is produced in much greater quantities (Yoneyama et al. 2020a) (**Fig. 1**). CLA may have been overrepresented in this assay due to the instability of 1ʹ-OH-MeCLA or alternatively may be a non-enzymatic by-product (Yoneyama et al. 2020a). Due to its instability, if 1ʹ-OH-MeCLA is the branching inhibitor or precursor, it would likely need to be immediately stabilized or further converted *in planta*. However, the fact that 1ʹ-OH-MeCLA has been detected in shoot tissues of arabidopsis supports the premise that it is somehow stabilized *in planta* (Yoneyama et al. 2020a). Because CLA is produced in greater quantities than 1ʹ-OH-MeCLA by LBO, future research needs to investigate the possibility that LBO functions as a demethylase to remove the methyl group from MeCLA to produce CLA. How this relates to the function of MeCLA and the synthesis of the shoot branching inhibitor needs to be determined.

Regardless, LBO function is important for the regulation of shoot branching/tillering, as the *lbo* mutant in arabidopsis has an increased branching phenotype, albeit one which is weaker than other mutants in the core SL biosynthesis pathway (Brewer et al. 2016), and altered expression of *LBO* impacts tillering in *Panicum virgatum* L. (switchgrass) (Yang et al. 2022). It is commonly proposed that counter-adaptations between parasitic weeds and host plants have driven the diversification of SL biosynthesis genes and exuded SLs. In contrast, *LBO* is highly conserved and often present as a single copy gene, perhaps suggesting that the biosynthesis of the SL specific to bud outgrowth regulation has not been influenced by a similar competitive evolutionary pressure.

The additive branching phenotype of the *lbo clamt* double mutant plants in arabidopsis and the intermediate branching phenotypes of the *lbo* and *clamt* single mutants compared to the wild type and mutants in the core SL biosynthesis pathway (Brewer et al. 2016, Mashiguchi et al. 2022) raise the possibility that *clamt* and *lbo* are required for the synthesis of different SL molecular structures that function as bud outgrowth inhibitors. This hypothesis could be tested by examining the branching phenotypes of shoots of reciprocal grafts between *clamt* and *lbo*. Alternatively, the moderate branching phenotype of *lbo* and additive phenotype of *clamt lbo* double mutants might be due to MeCLA having some minor branch-inhibiting activity (see SL perception section, MeCLA can interact with the SL receptor; Abe et al. 2014).

Current research points toward canonical SLs not being involved in shoot branching and instead that 1ʹ-OH-MeCLA or other hydroxylated structures produced by LBO that are yet to be identified act as the SL shoot branching hormone or are important for its biosynthesis (**Fig. 1**, Boxes B and C). In maize, the P450 ZmCYP706C37 can produce several non-canonical SLs, by converting CL to zealactol, and MeCLA to zealactone via their corresponding intermediates (Li et al. 2023) (**Fig. 1**). Intriguingly, z*mcyp706c37* mutants that had undetectable zealactol and severely depleted zealactone did not display an increased branching phenotype. Similarly, the maize z*mmax1b* (*cyp711A*) mutant did not exhibit an increased branching phenotype despite reduced zealactone in the root exudate (Li et al. 2023). By contrast, mildly increased branching occurs in the *ccd8* mutant in maize, suggesting that a maize SL still exerts some repression of branching despite the presence of a dominant *TEOSINTE BRANCHED1* (*TB1*, discussed later) allele (Guan et al. 2012, 2023, Li et al. 2023). Together, these findings suggest that the non-canonical SLs zealactol and zealactone do not function in branching inhibition (Li et al. 2023).

In *L. japonicus*, another 2OGDD enzyme is encoded by the gene *LOTUSLACTONE DEFECTIVE* (*LLD*), from a clade that is phylogenetically close to LBO. LLD is required for the synthesis of the non-canonical SL, lotuslactone (**Fig. 1**), but not 5-deoxystrigol (Mori et al. 2020a). Future research should quantify branching phenotypes of mutants in the synthesis of other non-canonical SLs (e.g. the *lld* mutant in *L. japonicus* that is deficient in the non-canonical SL, lotuslactone; Mori et al. 2020a), to determine if one or multiple non-canonical SLs function as the branching hormone.

It is still unclear whether a particular SL molecular structure(s) functions *in planta* as the shoot branching hormone. One significant roadblock in addressing this has been the difficulty in detecting SLs in shoot tissue. Indeed, early reports of SLs in shoot tissue found canonical SLs 5-deoxystrigol (in sorghum) and *epi*-5-deoxystrigol (in rice) to be at the limit of detection (∼2 pg·g^−1^ and <10 pg·g^−1^ fresh weight, respectively) (Yoneyama et al. 2007b, Umehara et al. 2010) and orobanchol and 4-deoxyorobanchol (in rice) to be below the limit of detection or absent in shoot tissue (Yoneyama et al. 2007b, Umehara et al. 2010; Xie et al. 2015b, Ito et al. 2022). However, as discussed earlier, it is likely that the SLs that more specifically regulate bud outgrowth *in planta* are not yet known, have non-canonical structures such as 1ʹ-OH-MeCLA or its downstream products and/or are produced only in specific tissues.

More recently, CL, CLA and MeCLA, in addition to various hydroxylated CL derivatives and hydroxylated CLA metabolites, have been detected in shoot tissue at levels comparable to those observed in root tissue (Yoneyama et al. 2020a, Mashiguchi et al. 2022) (**Fig. 1**). This suggests that they are important precursors to the SL shoot branching hormone. The identification of MeCLA, which is not a precursor to canonical SLs but is a precursor to many of the known non-canonical SLs (**Fig. 1**), in shoot tissue supports a role for non-canonical SLs in the shoot. Research is therefore needed to focus on non-canonical SL biosynthesis pathways downstream of, or parallel to, CLAMT and LBO. It will be interesting to discover if the SL molecular structure(s) that functions as the shoot branching hormone *in planta* is conserved across species or if there is diversity in the structure of the bioactive hormone across species, such as exists for gibberellin (Yamaguchi 2008).

Where are SLs produced in the plant? Expression studies indicate that SL biosynthesis genes are expressed throughout the plant, with vascular localization (Sorefan et al. 2003, Booker et al. 2005, Zou et al. 2006, Arite et al. 2007, Lin et al. 2009, Brewer et al. 2016). While expression in the roots is consistent with the role of SLs in the rhizosphere, shoot expression of biosynthesis enzymes is substantial. Indeed, it is often overlooked that grafting studies indicate that the shoot branching inhibitor can be produced in shoot tissue alone and that production in a small stem inter-graft segment is in fact sufficient to inhibit bud outgrowth at the nodes above the graft union (Foo et al. 2001, Simons et al. 2007). *CLAMT* and *LBO*, which are currently the last known enzymatic steps in the production of the SL shoot branching inhibitor, are both strongly expressed at the node, suggesting that the production of the shoot branching hormone occurs local to the axillary bud (Brewer et al. 2016, Mashiguchi et al. 2022). The presence of MeCLA in the shoot at levels comparable to root tissue (Yoneyama et al. 2020a) also suggests that the level of the SL shoot branching inhibitor in the shoot itself may be substantial. As current technologies for quantifying SLs rely on information on their molecular structure, the identification of new SLs and/or new approaches to detect unidentified SLs must remain a priority.

## Regulation of SL Levels by Nutritional and Other Hormonal Factors Involved in Shoot Branching

**Fig. 2:**
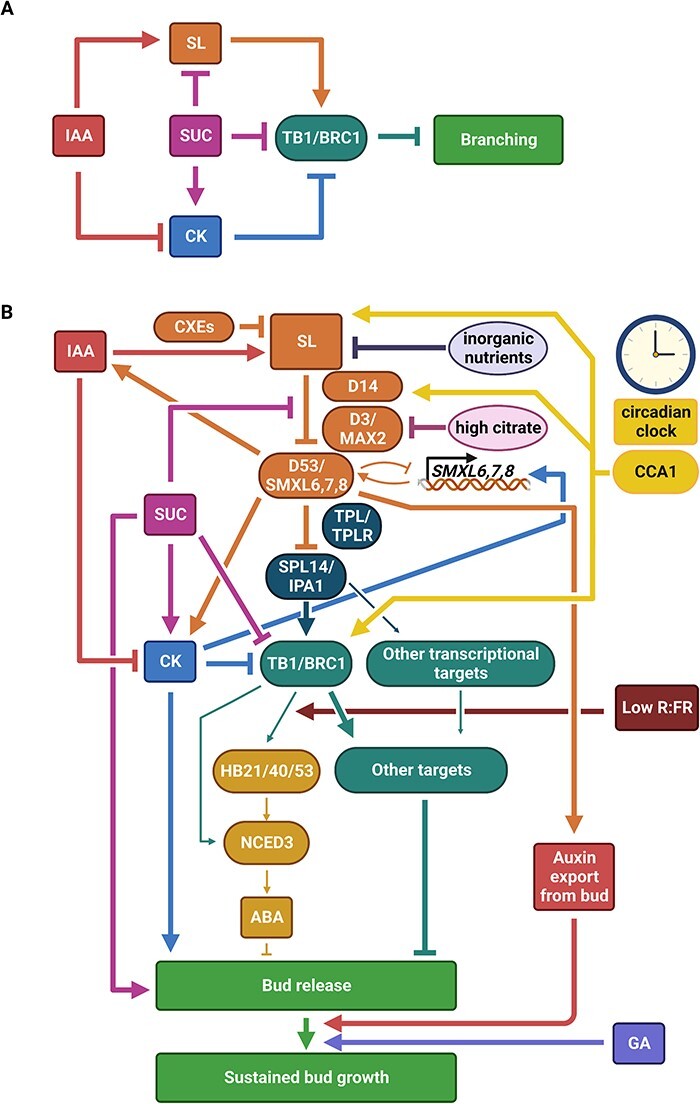
Simplified (A) and detailed (B) models for SL regulation of shoot branching. Branching is tightly regulated by the interplay of several signals. (A) The major regulators of branching are shown in simplified terms. Auxin (indole-3-acetic acid, IAA) inhibits branching by inducing SL and repressing cytokinin (CK). Sucrose (SUC) acts to promote bud release and can do so via repression of the SL pathway and promotion of the CK pathway. TB1/BRC1 is a conserved transcription factor regulated by SL, CK and SUC; SL enhances TB1/BRC1 and CK and SUC represses TB1/BRC1. TB1/BRC1 functions to inhibit branching. (B) Specific details of how SL interacts with other signals to regulate branching. SL biosynthesis is promoted by IAA and typically repressed by inorganic nutrients nitrate, phosphate and/or sulfate. SL is hydrolyzed by CXEs. D14 and D3/MAX2 are required for SL perception and signaling and are negatively perturbed by SUC and high citrate. SL perception and signaling target D53/SMXL6,7,8 proteins for degradation. An autoregulatory feedback loop exists whereby the SMXL6 protein represses transcription of SMXL6,7,8. D53/SMXL6,7,8 repressor proteins work together with TOPLESS (TPL) and TPL-RELATED (TPLR) proteins to suppress transcriptional activation by SPL14/IPA1 proteins. Following the degradation of D53/SMXL6,7,8, the SPL14/IPA1 can regulate TB1/BRC1 and other SL transcriptional targets. D53/SMXL6,7,8 also regulates IAA biosynthesis and CK metabolism; this has not yet been demonstrated to be via SPL14/IPA1/TPL/TPLR and so is shown independently in the figure. BRC1 appears to be the predominant target of this pathway and inhibits bud release. CCA1 is an important component of the circadian clock and regulates SL biosynthesis, SL signaling and TB1/BRC1. One minor pathway that is targeted by TB1/BRC1 is ABA—TB1/BRC1 promotes the expression of *HB21/40/53* and this is enhanced by low R:FR light, which promotes *NCED3*, leading to enhanced ABA content. BRC1 also regulates NCED3 independent of HB21, HB40 and HB53. ABA inhibits bud release. The major transcriptional target(s) of the SL and TB1/BRC1 pathways that account for the majority of bud outgrowth regulation is yet to be characterized. Following bud release, the bud can enter a state of sustained bud growth where it grows into a branch. Auxin export from the bud, which is reduced by SL via a non-transcriptional process, promotes the transition of a growing bud to a branch. Gibberellin (GA) enhances sustained bud growth. Arrowheads indicate promotion, and flat-ended lines indicate inhibition; line thickness is reduced in areas of the network that may have a comparatively less or restricted contribution to branching control. The figure was created with BioRender.com.

There are multiple points in the SL pathway that are regulated by other factors to regulate shoot branching, including regulation of SL levels and SL signaling. Regulation of SL levels is a common target of many nutritional and hormonal factors. For example, depending on plant species, nitrogen and/or phosphate availability promote shoot branching and tillering and this is presumed to be achieved at least in part via inhibition of SL biosynthesis, reducing SL levels (measured in the root and/or root exudate) (Yoneyama et al. 2007a, 2007b, 2012, López-Ráez et al. 2008, Umehara et al. 2008, Foo et al. 2013, de Jong et al. 2014, Sun et al. 2014, Barbier et al. 2023) (**Fig. 2**). This occurs via transcriptional regulation of SL biosynthesis genes as shown across many species (e.g. *D27, CCD7, CCD8*, various *CYP711A* subfamily members, *CYP722C, LLD* and *LBO*), including specifically at the node and in the bud (Xu et al. 2015, Abuauf et al. 2018, Wakabayashi et al. 2020, Mori et al. 2020a, Yoneyama et al. 2020b, R. Wang et al. 2020, Zha et al. 2022). Interestingly, sulfur deficiency in rice also enhances SL levels in the root and root exudate and this is correlated with a reduction of tiller bud outgrowth (Shindo et al. 2018) (**Fig. 2**). However, in the case of sulfur deficiency, this is associated with upregulation of *D27* expression, with only minor or no changes observed in the expression of other SL biosynthesis genes (Shindo et al. 2018). Future studies should quantify nutritional effects on SL precursors CL, CLA and MeCLA, and downstream SLs in shoot tissue, as indeed CL in root exudates is not increased by phosphate deficiency in rice (Seto et al. 2014).

The phytohormone auxin, produced in the shoot tip, has long been implicated in the inhibition of bud outgrowth due to its role in apical dominance, the process by which a growing shoot tip inhibits the outgrowth of axillary buds at the nodes below (Barbier et al. 2017, 2019, Beveridge et al. 2023). Consistent with its role in inhibiting bud outgrowth, auxin enhances the expression of SL biosynthesis genes *D27*, *CCD7*, *CCD8* and *LBO* (Sorefan et al. 2003, Foo et al. 2005, Arite et al. 2007, Hayward et al. 2009, Waters et al. 2012, Brewer et al. 2016, Abuauf et al. 2018) and SL levels (Foo 2013, Yoneyama et al. 2015) (**Fig. 2**). Cytokinin, a phytohormone that promotes bud outgrowth and tillering, represses SL levels in rice via transcriptional regulation of SL biosynthesis genes *OsD27, OsCCD7, OsCCD8, OsCYP711A2* and *OsCYP711A3*, and this is suggested to be independent of phosphate regulation (Yoneyama et al. 2020b). Gibberellin, a phytohormone that promotes sustained outgrowth of branches in pea (Cao et al. 2023) (**Fig. 2B**), has also been shown to repress SL (measured in root exudate) via transcriptional regulation of SL biosynthesis genes (*CCD7, CCD8, CYP711A9, DSD* and *LLD*) in *L. japonicus* (Mori et al. 2020a).

As SLs are carotenoid-derived, perturbations in the carotenoid pathway upstream of all-*trans*-β-carotene can also impact SL production. For example, a chloroplast-localized ζ-carotene isomerase (Z-ISO) catalyzes the isomerization of 9,15,9ʹ-tri-*cis*-ζ-carotene to form 9,9ʹ-di-*cis*-ζ-carotene, an intermediate upstream of all-*trans*-β-carotene, the precursor to both SL and abscisic acid (ABA) biosynthesis pathways (Liu et al. 2020). Accordingly, *tillering20* mutants that have impaired Z-ISO exhibit reduced ABA (measured in shoot bases and roots), reduced SL (4-deoxyorobanchol, measured in the root exudate) and an increased tillering phenotype that can be restored by exogenous application of SL or ABA (Liu et al. 2020). It is also likely that other cross talk exists between the ABA and SL biosynthesis pathways, as the hydroponic supply of ABA leads to the downregulation of expression of key SL biosynthesis genes in rice roots and reduced SL content in the root exudate after long-term ABA supply (Liu et al. 2020). Another important cross talk between the SL and ABA pathways will be discussed later.

Zaxinone, an apocarotenoid metabolite produced by a CCD subfamily member, is another endogenous regulator of SL synthesis. Mutants that are unable to produce zaxinone have elevated SL levels and enhanced expression of SL biosynthesis genes and both of which are restored by the exogenous supply of zaxinone (Wang et al. 2019).

The expression of SL synthesis genes is also under feedback regulation, leading to increased transcript levels of SL biosynthesis genes in SL-increased branching mutants (e.g. Foo et al. 2005, Johnson et al. 2006, Arite et al. 2007, Umehara et al. 2008, Drummond et al. 2009, Dun et al. 2009, Hayward et al. 2009, Brewer et al. 2016). This is thought to be mediated, at least in part, by *RAMOSUS2* which encodes an auxin receptor in the AUXIN-SIGNALING F-BOX4/5 (AFB4/5) clade (Ligerot et al. 2017). However, AFB4/AFB5 also show high affinity for binding picloram, a synthetic picolinate auxin, and further research should determine if there is an endogenous picolinate-like ligand specific to this clade of auxin receptors (Ligerot et al. 2017).

## SL Breakdown and Potential Sequestration

The recent discovery of carboxylesterases (CXEs) that hydrolyze SLs is an exciting advance and opens possibilities for dynamic and localized management of SLs and shoot branching phenotypes (Humphreys and Smith 2021, Xu et al. 2021, L. Wang et al. 2022) (**Fig. 2**). Interestingly, CXEs are in the same α/β-hydrolase superfamily as the SL receptor (discussed later). Due to their homology and production of the same reaction products, CXE15 and the SL receptor likely have the same reaction mechanism (Humphreys and Smith 2021, Xu et al. 2021). Grafting studies suggest that CXE activity in shoots might be important for the regulation of bud outgrowth, as wild-type rootstocks are unable to reduce branching in transgenic arabidopsis scions overexpressing *AtCXE15* (Xu et al. 2021). In addition, GUS staining assays using the *AtCXE15* promoter suggest that *AtCXE15* is expressed in multiple parts of the plant including shoot vasculature and the region of axillary buds (Xu et al. 2021). The expression of *AtCXE15* and its homologs in *Nicotiana tobacum* (tobacco) is regulated by SL, auxin and various environmental factors (Xu et al. 2021, L. Wang et al. 2022), suggesting this enzyme might be an important player in the environmental regulation of SLs and shoot branching.

While *in vitro* studies show CXE15 can hydrolyze diverse SLs including canonical SLs 5-deoxystrigol and orobanchol and the non-canonical SL MeCLA (Xu et al. 2021), the specificity of SL catabolism by CXE *in planta* needs to be determined. Additionally, further research is required into other CXEs, particularly CXE20. AtCXE20 was discovered in high-density planting and drought-tolerance activation tagging screens and its overexpression results in increased branching (Roesler et al. 2021, Simmons et al. 2021). 3D structural modeling with CXE20 has revealed that efficient SL hydrolysis by this protein is unlikely (Roesler et al. 2021). As CXE20 binds SLs, this indicates a potential role of CXE20 in SL stabilization and/or storage (Roesler et al. 2021).

## SL and SL Precursor Transport for Regulation of Branching

Hormones are often translocated over significant distances and/or to specific regions to elicit a response. Even before the identification of SLs as the shoot branching hormone, grafting studies with increased branching mutants demonstrated the long-distance, mobile nature of the hormone in petunia, pea and arabidopsis. Reciprocal grafting studies between wild-type and various SL mutant genotypes elegantly demonstrated the unidirectional movement of SLs from root to shoot to inhibit bud outgrowth (reviewed in Kameoka and Kyozuka 2018). Although grafting studies have demonstrated that long-distance transport occurs to regulate bud outgrowth, it is not clear if this is what normally occurs in plants. It is tempting to speculate that such long-distance regulation of bud outgrowth normally occurs *in planta* as a convenient method to communicate the nutrient status of the roots, e.g. to the growing shoot, and to modify growth accordingly.

The identity (or identities) of the mobile form(s) of SLs and their precursors are not fully clear. Mutant *max1* rootstocks are able to inhibit branching in *max4* mutant shoots in arabidopsis and as CL and hydroxy CLs are the biosynthetic intermediates between the MAX4 and AtCYP711A1 (MAX1) enzymes (Booker et al. 2005, Seto et al. 2014), this suggests that CL and/or hydroxy CLs are mobile (**Fig. 1**, Box A). Quantitative analyses have indeed confirmed that CL is mobile over long distances (Mashiguchi et al. 2022); the mobility of hydroxy CLs is yet to be confirmed quantitatively.

A product downstream of CL/hydroxy CLs must also be mobile since branching can be inhibited in *max1* mutant shoots by grafting to wild-type rootstocks (Booker et al. 2005) or *AtCXE15-OE* rootstocks (Xu et al. 2021). The downstream product(s) CLA, hydroxy CLAs and/or MeCLA might be translocatable from rootstock to scion as *clamt* or *lbo* mutant rootstocks can also reduce branching in *max1* scions (Brewer et al. 2016, Mashiguchi et al. 2022). Since CXE15 can hydrolyze MeCLA, CLA and/or hydroxy CLAs must be mobile from rootstock to shoot to explain the observed reduction in *max1* scion branching by *AtCXE15-OE* rootstocks (Xu et al. 2021) (**Fig. 1**). Furthermore, *clamt* rootstocks can repress branching in *max1* scions providing further support that the biosynthetic intermediate, CLA and/or hydroxy CLAs, is mobile (Mashiguchi et al. 2022). Importantly, while CL and CLA are mobile, they are not bioactive, do not bind to the SL receptor (see SL perception section) and require further conversion to be able to inhibit bud outgrowth (Abe et al. 2014, Brewer et al. 2016, Mashiguchi et al. 2022).

Wild-type rootstocks are able to reduce branching in *lbo* scions, an observation which would normally be interpreted as indicating that the product of LBO, 1ʹ-OH-MeCLA or other hydroxylated structures yet to be identified (**Fig. 1**, Boxes B and C), or a product further downstream, function as a long-distance signal (Brewer et al. 2016). However, wild-type rootstocks are unable to reduce branching in *clamt* scions, suggesting that MeCLA and downstream products are not mobile from rootstock to shoot (Mashiguchi et al. 2022). In contrast, wild-type rootstocks can reduce branching in *clamt lbo* double mutant scions (Mashiguchi et al. 2022). Since any variation in the rosette leaf number impacts the rosette branch number in highly branched arabidopsis genotypes (Fichtner et al. 2022), it needs to be determined if wild-type rootstocks can reduce rosette branching in *clamt* mutant scions when branching is measured as rosette branches per rosette leaf as seen in *lbo* (Brewer et al. 2016). If it is indeed the case that wild-type rootstocks are unable to inhibit branching in *clamt* scions, then these conflicting results suggest that CLA, but not downstream products, is mobile from root to shoot. They also suggest that the reduction of branching in the *lbo* scion by the wild-type rootstock may be due to a feedback upregulation of CLA from the wild-type rootstock that can then be converted to MeCLA in the *lbo* scion, but not the *clamt* scion, and then converted to alternative downstream SLs that have some level of bioactivity in *lbo*. While, as discussed later, it is possible that MeCLA itself might be bioactive, MeCLA treatment does not reduce branching in *lbo* mutant backgrounds (Brewer et al. 2016).

Exactly how SLs move throughout the plant to regulate bud outgrowth remains an open question confounded by the difficulty in quantifying SLs in plant shoot material (Yoneyama and Brewer 2021). One study has reported the detection of various SLs in the xylem sap of arabidopsis and tomato (Kohlen et al. 2011). However, subsequent studies have failed to detect known SLs or intermediates in xylem sap (Xie et al. 2015b).

The first identified SL transporter, PLEIOTROPIC DRUG RESISTENT 1 (PDR1), is an ATP-binding cassette (ABC) subtype G (ABCG) transporter that has a polar and asymmetric localization and was demonstrated to function as a cellular exporter of SL in petunia (Kretzschmar et al. 2012, Sasse et al. 2015). In addition to its role in facilitating SL exudation from roots, several lines of evidence suggest that PDR1 may be important for the transport of SLs within shoots to inhibit bud outgrowth. Increased shoot branching is observed in petunia *pdr1* mutant plants and in tobacco lines that have reduced expression of *PDR6*, a *PDR1* homolog, and the expression of *PDR1* is observed in stem vasculature and nodal tissue adjacent to leaf axils (Kretzschmar et al. 2012, Xie et al. 2015a). This is consistent with a role for PDR1 in transporting SLs and/or precursors in the shoot to a region near axillary buds. Interestingly, *PDR1* is expressed around the base of a dormant axillary bud (Shiratake et al. 2019), but expression is absent from the dormant axillary bud itself (Kretzschmar et al. 2012). Petunia grafting studies call into question the importance of PDR1 for long-distance SL transport from root to shoot, as *pdr1* rootstocks are able to transport sufficient SLs to inhibit branching in *decreased apical dominance 1* (*dad1*/*ccd8*) mutant scions (Shiratake et al. 2019). Instead, PDR1 is suggested to be important for short-distance cell-to-cell transport of SLs from vasculature toward the region of the axillary bud (Shiratake et al. 2019).

There are many unanswered questions about SL transport. While the importance of ABCG subfamily members for the transport of SLs is yet to be established across diverse species, the discovery of the root-specific ABCG59 in *Medicago truncatula* that is thought to be required for normal exudation of SL from the root into the rhizosphere supports a role for this family of proteins in SL transport across plant species (Banasiak et al. 2020). In addition, two genes encoding a maize homolog of PDR1 ABC transporter proteins are co-expressed with SL biosynthesis genes (Ravazzolo et al. 2019).

The discovery of an SL transporter invites the possibility for active directed regulation of hormone transport as an additional mode of regulation of bud outgrowth (and other SL-regulated processes). Future research needs to determine if the transport of SLs in shoot tissue by PDR1 is conserved across species and if (and how) SL is transported into axillary buds. In addition, the substrate specificity of PDR1 needs to be determined. Moreover, the mode of long-distance SL (and/or precursor) transport remains to be discovered.

## SL Perception, Signaling and Downstream Effects Important for Shoot Branching

The SL receptor, DAD2/DWARF14 (D14), is an α/β-hydrolase that signals and deactivates the hormone by hydrolytic degradation (Arite et al. 2009, Hamiaux et al. 2012, de Saint Germain et al. 2016, Yao et al. 2016, Shabek et al. 2018, Seto et al. 2019, Mashiguchi et al. 2021, Tal et al. 2022). The precise details of the function and timing of SL hydrolysis are an area of investigation. Canonical SLs such as 5-deoxystrigol and the synthetic SL GR24 have been shown to bind to D14 (Seto et al. 2019). The non-canonical SL intermediate MeCLA, but not CL or CLA, has also been shown to bind to D14 (Abe et al. 2014). However, the binding of MeCLA to D14 may not result in significant hydrolysis (Xu et al. 2021), and it is not known if the binding of MeCLA to D14 results in SL signal transduction. Future research needs to establish if MeCLA is less hydrolyzed by D14 than other SLs and if MeCLA induces SL signal transduction. Combined, these results indicate that diverse SLs can bind to D14 and this should be confirmed in future studies to help determine if the diversity of SLs found *in planta* can elicit equivalent or varied signaling responses.

The SL signaling mechanism is an area of intense research. Results to date are difficult to integrate into a universal model and are discussed in great detail in Mashiguchi et al. (2021). The binding of SL to D14 facilitates the interaction with the F-box protein DWARF3 (D3)/MAX2 (Yao et al. 2016, Hu et al. 2017, Shabek et al. 2018). There is conjecture surrounding the specifics of how SL binding to D14 induces the interaction with D3/MAX2. Early reports suggested a critical role for D14 hydrolysis of SL to promote the interaction between D14 and D3, whereby the hydrolysis product remained covalently linked to D14 (Yao et al. 2016). However, later reports demonstrated hydrolysis by D14 is not essential for SL signaling, and it was instead concluded that D14 hydrolysis of SLs occurs after signal transmission (Shabek et al. 2018, Seto et al. 2019). It has been proposed that D3 blocks D14 hydrolytic activity to prevent premature SL hydrolysis, as hydrolysis of SL is slowed when D14 is recruited by D3 (Yao et al. 2016, Shabek et al. 2018, Tal et al. 2022). Whether D14 can hydrolyze SL in the absence of D3 and D53 *in planta* remains to be determined, although the hydrolytic activity of D14 in *in vitro* enzymatic studies and in mutants that have lost receptor activity but retain hydrolase activity would suggest that it might (de Saint Germain et al. 2016, Yao et al. 2016, Xu et al. 2021).

The interaction of SL bound D14 with D3/MAX2 results in polyubiquitination and degradation by the 26S proteasome pathway of target repressor protein DWARF53 (D53) and its orthologs SUPPRESSOR OF MAX2 1-LIKE6, 7 and 8 (SMXL6, SMXL7 and SMXL8) (Jiang et al. 2013, Zhou et al. 2013, Soundappan et al. 2015, Yao et al. 2016, Shabek et al. 2018, Kerr et al. 2021) (**Fig. 2**). D53, SMXL6, SMXL7 and SMXL8 proteins inhibit SL signaling by repressing transcription of SL targets. They interact with TOPLESS (TPL) and TOPLESS-RELATED (TPLR) proteins and certain SQUAMOSA PROMOTER-BINDING PROTEIN-LIKE (SPL) proteins to suppress the transcriptional activation activity of SPLs (Jiang et al. 2013, Zhou et al. 2013, Smith and Li 2014, Soundappan et al. 2015, Wang et al. 2015, Liu et al. 2017, Song et al. 2017, Xie et al. 2020, Sun et al. 2021) (**Fig. 2**). SMXL6 also directly binds to DNA, including the promoter region of *SMXL6, SMXL7* and *SMXL8*, and suppresses the expression of downstream genes (L. Wang et al. 2020). This negative autoregulatory feedback likely functions to maintain and restrict SMXL protein levels allowing for dynamic regulation of bud outgrowth, while also maintaining SL signaling homeostasis and preventing unrestrained branching.

As a further part of the mechanism of SL homeostasis, SL signaling induces the ubiquitination and degradation of the SL receptor, D14 (Chevalier et al. 2014, Hu et al. 2017, Tal et al. 2022). One model for SL signaling suggests that a conformational change of D2/MAX2 is important (Tal et al. 2022). Following ubiquitination of D53/SMXL6,7,8, D14 hydrolysis of SL leads to D14 topological changes and along with D3/MAX2 conformational changes, leads to removal of D53/SMXL6,7,8 allowing ubiquitination of D14 and proteasomal degradation (Tal et al. 2022).

A potentially important cross talk between the SL and gibberellin pathways (Sun et al. 2023) has recently been revealed in relation to nutrient responses. It was demonstrated that under high nitrogen supply, D53 and a negative regulator of gibberellin signaling, the DELLA protein SLENDER RICE1, bind the transcription factor GROWTH-REGULATING FACTOR4 to prevent its transcriptional activation of downstream nitrogen response genes (Sun et al. 2023). Low-nitrogen conditions, which increase SL levels, therefore reduce levels of SLR1 and D53, which allows the GRF4 transcription factor to bind and activate transcription of low-nitrogen response genes.

An important transcriptional target for the regulation of bud outgrowth by the SL pathway is *TB1*/*FINE CULM1*/*BRANCHED1* (*BRC1*), which encodes a TB1, CYCLOIDEA, PCF (TCP) transcription factor that is predominantly expressed in axillary buds and represses their outgrowth (Aguilar-Martínez et al. 2007, Braun et al. 2012, Dun et al. 2012, Soundappan et al. 2015, Wang et al. 2015, 2020b, Liu et al. 2017) (**Fig. 2**). Transcriptomic analyses have revealed about 400 SL responsive genes in arabidopsis (L. Wang et al. 2020) and many putative TB1/BRC1 targets (González-Grandío et al. 2017, Dong et al. 2019). One such example links TB1/BRC1 with ABA. Under short photoperiods or low R:FR light, BRC1 induces transcription of three related homeodomain leucine zipper protein transcription factors [*HOMEOBOX PROTEIN 21* (*HB21*), *HB40* and *HB53*], which then, together with BRC1, enhance the expression of *9-CIS-EPOXICAROTENOID DIOXIGENASE 3* (*NCED3*), which in turn leads to local accumulation of ABA (González-Grandío et al. 2017) (**Fig. 2**). The regulatory module from BRC1 through to ABA to inhibit bud/tiller outgrowth appears conserved, as it is also present in maize (Dong et al. 2019). This begs the questions of whether SL regulates ABA via BRC1 or this is an SL-independent BRC1 effect and whether this is an important mechanism for SL regulation of bud outgrowth. Indeed, for many species, there is a correlation between ABA content in buds and bud dormancy (Pan et al. 2021).

A few lines of correlative evidence support SL regulation of ABA for the regulation of bud outgrowth: (I) BRC1-dependent, SL induction of *HB40* is observed under normal light conditions (L. Wang et al. 2020); (II) ABA is reduced in SL-deficient mutant shoot bases (rice) and SL-deficient and *brc1* mutant buds in arabidopsis (Liu et al. 2020, L. Wang et al. 2020); (III) ABA levels are modestly increased in the inhibited *smxl678* triple mutant buds in arabidopsis (L. Wang et al. 2020); (IV) treatment of 3 µM ABA to rice SL mutants can repress their increased tillering phenotype (Luo et al. 2019) and (V) overexpression of *NCED1* in rice leads to a substantial increase in ABA content accompanied by a decrease in the tiller number (Luo et al. 2019).

While the above is good correlative evidence for SL and BRC1 regulation of ABA for the regulation of bud outgrowth, further evidence is required to unequivocally demonstrate conserved causality across diverse species. It needs to be demonstrated across diverse species that ABA content in buds of SL mutants is reduced and that ABA treatment to SL mutant buds can inhibit their growth at physiologically relevant concentrations. Furthermore, if ABA were a major target of the SL pathway to regulate bud outgrowth, it would be expected that ABA mutants would display altered bud outgrowth phenotypes similar to SL mutants under normal light conditions, and this deserves further investigation. Indeed, rice ABA biosynthesis mutants show increased tillering at upper nodes (Liu et al. 2020); however, this is not consistent with the increased tillering observed at basal nodes of SL mutants. Additionally, ABA biosynthesis mutants in arabidopsis have modest increases in branching under both low and high red:far red light conditions (Reddy et al. 2013, Yao and Finlayson 2015). While the relatively minor increase in rosette branching in the *hb21 hb40 hb53* triple mutant compared to the wild type might indicate that it is unlikely that ABA is a major target of the SL pathway, this could be a result of BRC1 regulating *NCED3* expression independent of HB21, HB40 and HB53 (González-Grandío et al. 2017) (**Fig. 2**). It may be difficult to disentangle individual hormone effects on bud outgrowth due to the cross talk between the SL and ABA biosynthesis pathways (Liu et al. 2020). For example, ABA mutants need to be tested to identify whether they have a reduced response to SL treatment. The proportion of SL inhibition of shoot branching that is mediated by ABA as compared with other pathways is therefore yet to be established (**Fig. 2**).

SL probably also regulates the bud content of the branch stimulatory hormone cytokinin. This SL effect involves transcriptional modulation of cytokinin biosynthesis (in pea) and metabolism (in pea and rice) (Duan et al. 2019, Zha et al. 2022, Cao et al. 2023) (**Fig. 2**). SL regulation of bud cytokinin is likely to be D53/SMXL6,7,8-dependent due to the requirement of D53 for SL transcriptional regulation of *CKX9* in rice and the elevated bud cytokinin observed in the *d53* gain-of-function mutant shoot base (Duan et al. 2019). Further investigation is required across species to establish the dependence of SL regulation of bud cytokinin content on D53/SMXL6,7,8 and BRC1, and the importance of bud cytokinin for SL-mediated regulation of outgrowth, as cytokinin content and expression of biosynthesis genes are not consistently elevated in SL-increased branching mutant buds (Dun et al. 2012, Young et al. 2014).

The regulation of auxin transport is a non-transcriptional target of the SL pathway (Crawford et al. 2010, Shinohara et al. 2013, Soundappan et al. 2015, Zhang et al. 2020) (**Fig. 2**). SLs repress PIN-FORMED (PIN) proteins independently of BRC1 (van Rongen et al. 2019). Although this effect of SLs on PIN accumulation and auxin transport is not BRC1 dependent, it is dependent on SMXL6,7,8 (Soundappan et al. 2015). Specifically, SLs disrupt auxin feedback on PIN polar membrane localization and clathrin-dependent endocytosis of PIN proteins (Zhang et al. 2020). This process dampens new auxin canalization and reduces subsequent vasculature connections by decreasing the sink strength of existing auxin transport and vascular channels (Crawford et al. 2010, Shinohara et al. 2013, Zhang et al. 2020). This process inhibits buds from establishing auxin flow into the vascular bundles of the main stem. This is evident from altered vascularization patterns in the stem and leaves (Zhang et al. 2020) and in branches of the arabidopsis *max4* SL-deficient mutant that more frequently merge with stem vascular bundles and less frequently merge with leaf trace compared to those of wild-type plants (Ongaro et al. 2008).

Activation of auxin export from buds does not seem to be an initial trigger for bud outgrowth and instead is a complementary mechanism for SLs to modulate ongoing branch growth (Brewer et al. 2015, Barbier et al. 2019, Chabikwa et al. 2019, Cao et al. 2023) (**Fig. 2**). It seems auxin flow from buds is important for stimulating sustained bud outgrowth. Having SL to regulate this process provides a secondary mechanism for SLs to dynamically regulate bud and branch growth. As the process of auxin canalization relies upon the production of auxin, it needs to be tested if SL can repress auxin biosynthesis in the bud as it does in the stem, where SL treatment represses transcription of auxin biosynthesis genes and auxin content (Ligerot et al. 2017).

Like SL biosynthesis discussed earlier, SL signaling is another point of regulation by other factors that control branching. Recent advances have highlighted the important role of sugars as initial regulators of bud outgrowth via regulation of *BRC1* and cytokinin (Mason et al. 2014, Barbier et al. 2015, 2021, Fichtner et al. 2017, Bertheloot et al. 2020, Salam et al. 2021, Wang et al. 2021, Patil et al. 2022) (**Fig. 2**). Importantly, sucrose antagonizes SL suppression of bud outgrowth/tillering via regulation of *D3*/*MAX2* expression to suppress SL signaling (Patil et al. 2022, Barbier et al. 2023) (**Fig. 2**). Sucrose suppresses the rate of degradation of D53 by SL and greatly reduces SL-induced degradation of the SL receptor, D14 (Patil et al. 2022). Another metabolite, citrate, which is a highly abundant carboxylate within the Krebs/tricarboxylic acid cycle in plants (and animals), has recently been shown in vitro to impact D3/MAX2 activity (Tal et al. 2022). It is suggested that citrate can trigger the reopening of the D3 C-terminal helix (CTH), which is required for the D3–D14–D53 complex to form. However, high levels of citrate prevent the reclosing of the CTH, preventing the release of ubiquitinated D53 for degradation, thereby inhibiting SL signaling (**Fig. 2B**). This highlights another potential mechanism for plant carbon status to regulate SL signaling to control bud outgrowth and hence shoot architecture (Barbier et al. 2023). However, these observations are mainly based on in vitro experiments, and more work needs to be done to test this *in planta*.

In addition to cross talk on transcriptional regulation of SL biosynthesis, cytokinin and nitrogen also affect the expression of SL signaling genes. Cytokinin upregulates the expression of *SMXL7/D53* transcripts in buds (Kerr et al. 2021). The transcription factor NITROGEN-MEDIATED TILLER GROWTH RESPONSE 5 is required for the promotion of tillering by nitrogen, and this is mediated via transcriptional regulation of *D14, D3, SPL14* and *TB1* (Wu et al. 2020).

The circadian clock is an important regulator of plant growth and development, including flowering. While the underlying molecular basis for the flowering phenotypes of circadian clock mutants has been well studied, the molecular basis for their altered branching/tillering phenotypes has not yet received much attention. One important regulator of the circadian clock is the transcription factor CIRCADIAN CLOCK ASSOCIATED1 (CCA1). Interestingly, recent studies reveal that the circadian clock regulates tillering via transcriptional regulation of SL biosynthesis, response and signaling (F. Wang et al. 2020). Hormone treatment studies and double mutant analyses support a model where the circadian clock alters SL response and signaling to regulate tillering, as *cca1* mutants have increased tillering and are insensitive to inhibition of tillering by SL, and the increased tillering phenotypes of *cca1 d14* and *cca1 tb1* double mutants do not differ from the respective single mutants. It is likely that CCA1 is also an important integrator of sugar for the regulation of tillering, as functional CCA1 is required for response to changed sugar in both tillering phenotype and *TB1* expression (F. Wang et al. 2020). Future research should further explore the connections between flowering and branching regulation, as important targets for regulating plant overall growth and reproductive strategy.

Where do SL perception and signaling occur for the regulation of bud outgrowth? Shoot localization studies indicate that D14, MAX2 and D53/SMXL6,7,8 are all vascular localized (Stirnberg et al. 2007, Zhou et al. 2013, Chevalier et al. 2014, Soundappan et al. 2015). However, the exact cellular identity is unclear; D14 appears localized to the phloem (Chevalier et al. 2014, Kameoka et al. 2016), MAX2 is present in the phloem, cambium and xylem parenchyma (Stirnberg et al. 2007) and D53 appears localized to parenchyma cells surrounding the xylem (Zhou et al. 2013). The necessity of this shoot vascular localization for the regulation of bud outgrowth specifically is unknown, as MAX2 is required in the vascular cambium for SL signaling–mediated regulation of secondary growth (Agusti et al. 2011). MAX2, D14 and D53/SMXL6,7,8 are expressed in axillary buds (Stirnberg et al. 2007, Zhou et al. 2013, Soundappan et al. 2015, Kameoka et al. 2016, Katyayini et al. 2019), and studies with chimeric plants demonstrate that MAX2 is required locally (in or close to the axillary bud) to inhibit outgrowth (Stirnberg et al. 2007). Combined, it is likely that SL perception and downstream signaling occur at the bud to regulate local bud outgrowth.

Intriguingly, the D14 protein is present in phloem sap (Aki et al. 2008, Batailler et al. 2012), consistent with its expression specifically in phloem companion cells and sieve elements (Kameoka et al. 2016) and the observed short-distance mismatch in the localization of D14 mRNA and protein (Chevalier et al. 2014, Kameoka et al. 2016). Further research is needed to determine the significance of D14 transport in the phloem and whether it relates to its SL receptor and/or hydrolase activity or otherwise. Indeed, while D14 hydrolysis of SL is less efficient than hydrolysis by CXE15 (Xu et al. 2021), it is possible that D14 sequesters or safely transports and/or hydrolyzes SL in the phloem as another means of regulation.

## Future Perspectives and Challenges Ahead

Arguably, the main outstanding question here is the structural identity of the endogenous SL shoot branching hormone. This is clearly hampered by the instability of SLs and the difficulty in synthesizing them. As discussed, recent detailed enzymatic studies combined with mutant phenotypic analyses are ruling out specific SL molecular structures. Ultimately, the breakthrough identification of the shoot branching hormone structure and the characterization of the enzymatic reactions in its synthesis will present exciting opportunities to specifically manipulate shoot branching in crops to optimize yield without disruption to rhizosphere functions of SLs or, potentially, other important *in planta* functions. This is because it is likely that further enzymes that are required specifically for the synthesis of the branching hormone, but not root and rhizosphere canonical SLs, will be identified. These enzymes, such as CLAMT and LBO, are exciting targets to specifically manipulate bud outgrowth without as many effects on other aspects of development. While current research points to 1ʹ-OH-MeCLA (or other hydroxylated structures yet to be identified; **Fig. 1**, Boxes B and C), as the best candidate(s) for the shoot branching hormone or its precursor, the lability of 1ʹ-OH-MeCLA does beg the question of whether it is sufficiently stable to be an intermediate or active in its own right. It should be investigated if 1ʹ-OH-MeCLA can bind to, and be protected by, an α/β-hydrolase such as CXE20, which is proposed to bind and sequester but not hydrolyze SLs (Roesler et al. 2021).

One intriguing possibility for LBO function relates to the relative membrane permeability of CLA and MeCLA (**Fig. 1**). Due to being methylated, MeCLA is reasonably hydrophobic and would cross membranes more easily than CLA, which is hydrophilic due to its charged carboxylate group. Transport across membranes might be required for the SL inhibitor to be produced at the site of SL perception (e.g. within an axillary bud). If this is found to be the case, then the functions of CLAMT and LBO may be to modulate the conversion between transport (MeCLA) and precursor forms (CLA) of the SL branching inhibitor. That is, CLAMT might convert CLA to MeCLA to facilitate transport across membranes, and then once in contact with the axillary bud–expressed LBO enzyme, MeCLA would be converted back to CLA and be available for conversion to the bioactive SL branching hormone in the bud. This might explain why wild-type rootstocks are not able to suppress branching in *lbo* mutant shoots as effectively as for other SL biosynthesis mutant shoots, perhaps due to a limited ability of CLA to enter the axillary bud to be further converted to the unknown bioactive SL branching inhibitor (Brewer et al. 2016, Mashiguchi et al. 2022).

Continued research is required for the purpose of the structurally diverse SLs, canonical and non-canonical, both within and between plant species. Tightly controlled enzymatic regulation may enable spatial and temporal regulation of SL types and different SL-regulated processes, with the production of particular SL structures targeted to required localities. As such, the roles of canonical SLs beyond the rhizosphere and of specific non-canonical SLs such as lotuslactone (Mori et al. 2020b) that are unlikely important for the inhibition of branching need to be distinguished from SLs that are involved in shoot branching.

Finally, it is becoming clear that the SL pathway is an important integrator of nutritional and metabolic signals (Barbier et al. 2023, Beveridge et al. 2023). This should be a focus of future research, including potential roles in integrating reproductive strategy, as it has been shown that the reproductive strategy, circadian clock and flowering time affect branching (Beveridge et al. 2003, Fichtner et al. 2022, F. Wang et al. 2020).

## Data Availability

No new datasets were generated or analyzed for this review.
